# Research progress on the drug resistance mechanisms of *Candida tropicalis* and future solutions

**DOI:** 10.3389/fmicb.2025.1594226

**Published:** 2025-05-12

**Authors:** Huisheng Xiong, Rigetu Zhao, Shizhe Han, Zhilin Liu, Xin Zhang, Zelin Jia, Jiayu Cui, Yuhang Zhang, Xueli Wang

**Affiliations:** ^1^College of Animal Science and Technology, Inner Mongolia Minzu University, Tongliao, Inner Mongolia, China; ^2^Chifeng Agricultural and Animal Husbandry Science Research Institute, Chifeng, Inner Mongolia, China; ^3^Tongliao Naiman Banner Animal Disease Control and Prevention Center, Tongliao, Inner Mongolia, China

**Keywords:** *Candida tropicalis*, drug resistance mechanisms, azoles, polyenes, echinocandins, 5-fluorocytosine, antifungal candidates

## Abstract

*Candida tropicalis* is an important member of the non-*Candida albicans* species. It is closely associated with candidemia, especially common in neutropenic and critically ill patients. Drug-resistant *C. tropicalis* isolates have been found not only in clinical patients but also in animals, fruits, and the environment. In recent years, the detection rate of azole-resistant *C. tropicalis* isolates has increased. Drug-resistant *C. tropicalis* is related to persistent, recurrent, and breakthrough infections. Therefore, understanding its drug resistance is crucial for clinical treatment. The review explores the main mechanisms of *C. tropicalis* resistance to antifungal drugs and discusses the genetic basis involved in the antifungal resistance of *C. tropicalis*. In addition, current research on natural extracts, nanomaterials, etc. used for antifungal purposes has also been reviewed. The aim of this review is that in-depth research on the drug resistance mechanisms of *C. tropicalis* resistant strains can help guide clinical medication. Meanwhile, it can also provide new ideas for opening up new pathways, searching for new targets, and screening out safe and effective “antifungal candidates,” with the expectation of improving the current clinical cure rates.

## 1 Introduction

Fungi are a heterogeneous group of eukaryotic organisms and are one of the most widely distributed organisms on Earth. They include molds, yeasts, mushrooms, and other well-known fungi. Among the genus Candida, the pathogenic agent of infection is usually *Candida albicans* (*C. albicans*). Although *C. albicans* is a common dominant strain, the isolation rate of non-*Candida albicans* has increased significantly in recent years (Sadeghi et al., 2018). In particular, *Candida tropicalis* (*C. tropicalis*) ranks second among non-*C. albicans* species causing candidal infections (Smith and Rickerts, 2017). Invasive candidiasis (IC) is a severe infectious disease caused by several Candida species and is the most common mycosis in hospitals. The global prevalence is 250,000 to 700,000 people per year, the incidence rate is 2–14 cases per 100,000 people, and the mortality rate is 40–55% (Bongomin et al., 2017; Logan et al., 2020). The most common Candida species are *C. albicans*, *Candida glabrata*, *Candida krusei*, *C. tropicalis*, and *Candida parapsilosis*. It is worth noting that in recent years, there have been successive reports of animal-source IC (Seyedmousavi et al., 2018). For example, in birds, *C. albicans* infection can lead to raptor pulmonary candidiasis, chicken cutaneous candidiasis, and canary myocarditis. In dogs, there is peritonitis caused by *C. albicans* and *C. glabrata*; dermatitis, otitis externa, and urinary tract infections caused by *C. albicans* and *C. tropicalis*. In cats, urinary tract infections, intestinal granulomas, and empyema are caused by *C. albicans* and others. In ruminants, for example, mastitis and gastrointestinal infections in dairy cows caused by *C. albicans* infection, and abortions in pregnant female animals caused by *C. tropicalis*, etc. In addition, it has been reported that the susceptibility of *C. tropicalis* isolated from several animals to antifungal drugs has decreased (Álvarez-Pérez et al., 2016; Subramanya et al., 2017; Glushakova and Kachalkin, 2024). Moreover, the diagnosis and treatment of IC are a clinical challenge because traditional diagnostic methods such as isolation and culture lack high sensitivity and/or specificity (Lass-Flörl et al., 2021).

Moreover, due to the inherent and/or acquired resistance of the genus Candida, drug interactions, toxicity, and unpredictable pharmacokinetics, decisions regarding antifungal treatment and dosing can be complicated (Pappas et al., 2018). Azole antifungal drugs are commonly used for the treatment of Candida infections, among which fluconazole is the most widely used due to its low cost and few side effects (Ou et al., 2017; Wiederhold, 2017). However, with the increase in the incidence of *C. tropicalis* infections, a synchronous increase in the resistance of *C. tropicalis* to fluconazole has also been observed (Fan et al., 2017; Wu et al., 2017). It is worth noting that compared with *C. albicans*, *C. tropicalis* isolates are generally less sensitive to fluconazole and are more likely to develop azole resistance rapidly. Recent studies have shown that in the past decade, the antifungal resistance of clinical *C. tropicalis* isolates to azole drugs has increased at an alarming rate (Xiao et al., 2020; Wang Y. et al., 2021; Keighley et al., 2024). For example, a study in China showed that from August 2009 to July 2018, the resistance rates of *C. tropicalis* to voriconazole and fluconazole increased from 5.7 and 5.7% to 29.1 and 31.8%, respectively (Wang Y. et al., 2021).

Revealing the specific causes of drug resistance from the biological characteristics of *C. tropicalis*: Studies have shown that an important virulence factor of *C. tropicalis* is related to biofilm formation (Staniszewska, 2020). It increases immune evasion by forming biofilms (Cangui-Panchi et al., 2023). The *C. tropicalis* biofilm is a highly dense three-dimensional network system. The outer layer is the extracellular matrix, and the inside contains three cell forms: spores, hyphae, and pseudohyphae (Guo and Li, 2021). This structure can prevent the penetration of antibacterial drugs, evade the immune killing effect of the body, and promote the regeneration of the biofilm (Cavalheiro and Teixeira, 2018), making the infections caused by *C. tropicalis* more severe. *C. tropicalis* has a strong and rapid biofilm formation ability (Sahal and Bilkay, 2018; Lass-Flörl et al., 2024). Studies have shown that the bloodstream infections it causes are extremely prone to form biofilms, with a proportion as high as 70–90%, which is much higher than that of other Candida species (Atiencia-Carrera et al., 2022). Currently, some studies believe that the drug resistance mechanism of *C. tropicalis* is related to this special virulence factor of the biofilm, and it can increase the resistance of the strains to antifungal drugs by forming biofilms (Sasani et al., 2021b). In further studies, it was found that five genes, namely *ALS1*, *ALS2*, *BCR1*, *EFG1*, and *WOR1*, are involved in the biofilm formation process of *C. tropicalis* (Yang, 2023). In addition, studies on *C. tropicalis* from different ecological niches have shown that different ecological niches often affect its biological characteristics. In order to compare the biological characteristics of environmental isolates and commensal isolates, researchers explored their growth status under different stress conditions, the ability to produce secreted aspartyl proteases (Saps), and the ability to form hyphae. It is worth noting that filamentous growth is another virulence factor of the genus Candida (Hu et al., 2023). The study by Hu et al. (2023) showed that compared with environmental strains, commensal isolates showed no significant differences except for a stronger filamentous growth ability. This result indicates that commensal isolates may be more adaptable to the host’s microenvironment and may increase their ability to cause infections by enhancing invasive growth (Hu et al., 2023). As a result, a large number of *C. tropicalis* infection cases have emerged clinically. Subsequently, due to the widespread and irregular use of antifungal drugs, the drug resistance of *C. tropicalis* has been further exacerbated. In addition, *C. tropicalis* is considered a microorganism resistant to osmotic pressure. This ability to survive in high concentrations of salt may play an important role in the persistence of the fungus in saline-alkali environments, contributing to the expression of virulence factors *in vitro* and resistance to antifungal drugs (Zuza-Alves et al., 2016).

**Figure 1:**
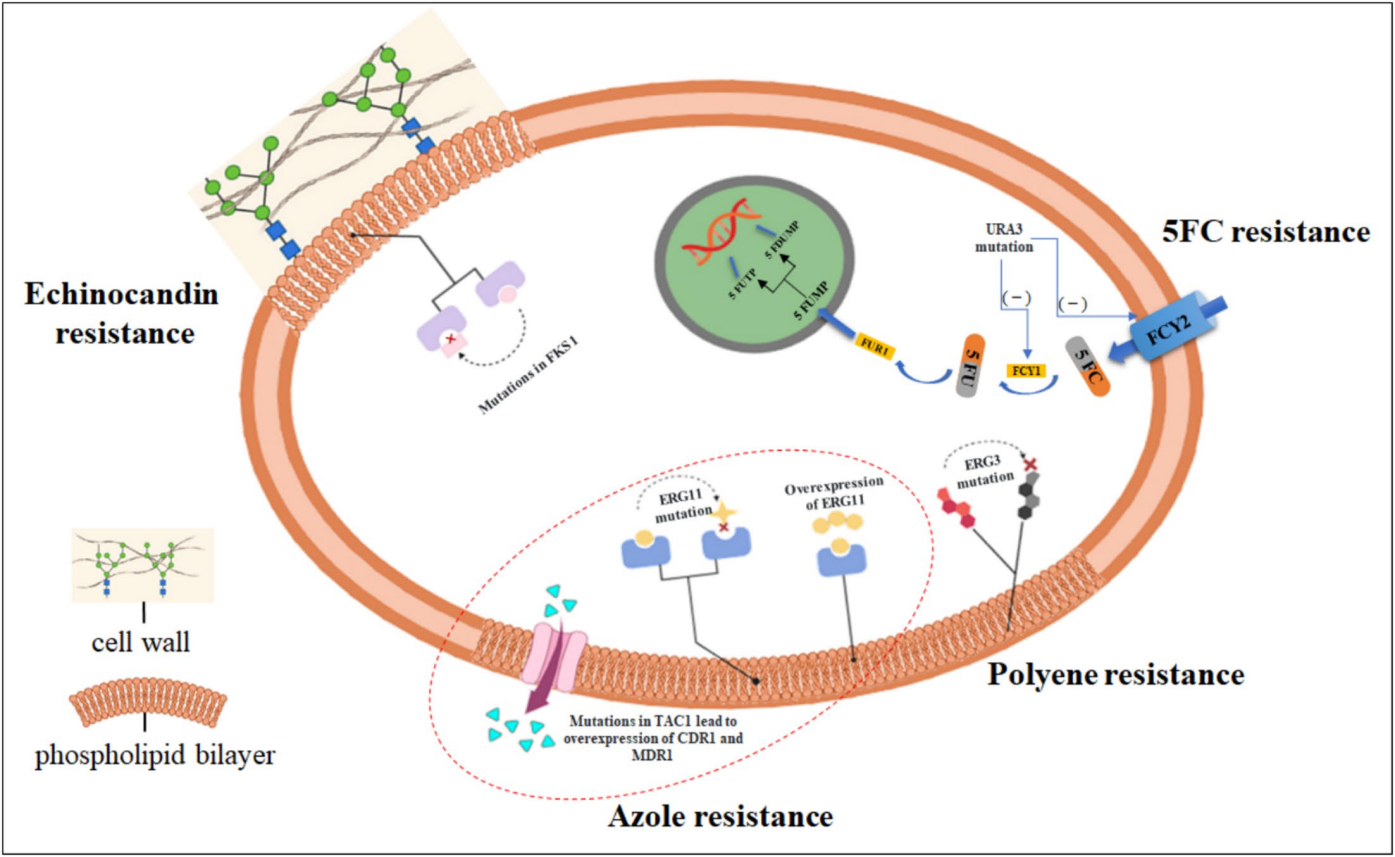
The main mechanisms by which *Candida tropicalis* develops drug resistance. Currently recognized resistance mechanisms of drugs against *C. tropicalis*. The most commonly observed resistance mechanism to azoles is the reduced intracellular accumulation of drugs through the overexpression of efflux pumps (for example, mutations in the *TAC1* gene lead to the overexpression of *CDR1* and *MDR1*); mutations in the *ERG11* gene can impede the inhibitory effect of azole drugs; gain-of-function mutations and overexpression in the *UPC2* gene result in the overexpression of the *ERG11* gene. Resistance to polyene drugs is due to the fact that mutations in the ergosterol biosynthesis gene *ERG3* lead to the accumulation of some alternative membrane sterols that do not interact with polyene drugs. Resistance to 5-fluorocytosine (5FC) is caused by metabolic disorders due to enzymatic mutations [for example, mutations in the *URA3* gene lead to a reduction in cytosine permeases (FCY1, FCY2)]. Resistance to echinocandins is almost entirely caused by point mutations in the hotspot regions of the *FKS1* gene. (Created in https://BioRender.com).

According to some recent research reports, *C. tropicalis* is resistant to currently available antifungal drugs such as azole derivatives, amphotericin B, and echinocandins (Schikora-Tamarit and Gabaldón, 2024; Díaz-García et al., 2023; Dawoud et al., 2024). This article reviews the mechanisms of azoles, echinocandins, polyenes, and 5-fluorouracil in treating *C. tropicalis* infections, as well as the mechanisms of the increased resistance of *C. tropicalis* to azoles, echinocandins, polyenes, and 5-fluorouracil. The main mechanisms of the increased resistance of *C. tropicalis* to antifungal drugs are shown in Figure 1. Since the widespread use of antifungal drugs such as azoles to treat *C. tropicalis* infections has led to an increase in its resistance, it is crucial to have antifungal drugs that can treat *C. tropicalis* infections without causing an increase in resistance. Through a comprehensive analysis of a large number of literatures, this review reveals some natural extracts and chemical substances with potential against drug-resistant *C. tropicalis*, in the hope of providing ideas for solving the increasingly serious problem of *C. tropicalis* resistance. Moreover, for the first time, it comprehensively summarizes the structural and functional differences of calcineurin among different Candida species, and how these differences affect drug tolerance, providing an important theoretical basis for the development of specific treatment strategies for *C. tropicalis*.

## 2 Resistance mechanism to azole drugs

Azole antifungal drugs (isavuconazole, posaconazole, voriconazole, and fluconazole) are the broad-spectrum antifungal drugs most commonly used in clinical practice. Their mode of action is to bind the nitrogen atom of the drug itself to the heme iron of cytochrome P450 in 14*α*-demethylase, blocking the incorporation of ergosterol and leading to the accumulation of the synthesis precursor 24-methylene dihydrolanosterol. These intermediate sterols do not have the same configuration and physical properties as ergosterol. Therefore, they will cause changes in the properties of the plasma membrane, such as altering fluidity, permeability, and impairing nutrient absorption, ultimately leading to cytotoxicity (Cowen, 2008). Ergosterol is an important component of the fungal cell wall, and lanosterol 14-α-sterol demethylase is involved in the biosynthesis of ergosterol, so it is a promising antifungal target (Chong et al., 2018). Therefore, azole drugs bind to 14α-demethylase, block the conversion of lanosterol to ergosterol, cause changes in the structure and function of the cell membrane, inhibit fungal growth, and thus achieve the therapeutic effect on fungal infections. However, due to the affordable price of azole drugs, their widespread use has led to a certain degree of drug abuse (Wiederhold, 2017). Since they only have an inhibitory effect on pathogenic fungi but no bactericidal effect, the problem of Candida resistance to azole drugs is becoming increasingly serious (Xu and Yan, 2023). In addition, there are numerous examples of azole resistance. For instance, studies by Ramos et al. (2024), Bilal et al. (2023), and Medeiros et al. (2025) have all demonstrated that *C. tropicalis* is resistant to azoles. It is worth noting that some studies have confirmed that the abundance of carbon substrates (glucose, fructose, and sucrose) can affect or enhance the resistance of *C. tropicalis* to fluconazole and its tolerance to osmotic pressure and oxidative stress (Khamrai et al., 2024).

### 2.1 Mutations or/and overexpressions of genes in the ergosterol synthesis pathway

*ERG11*, also known as *CYP51* (encoding lanosterol 14α-demethylase), is a member of the cytochrome P450 family and is a target of azole antifungal drugs (de Barros et al., 2018; Sasani et al., 2021a). Azole drugs inhibit the biosynthesis of ergosterol by binding to fungal cells, which may lead to drug resistance (Whaley et al., 2017; Jiang et al., 2024). Studies have shown that the main mechanism of azole resistance in the genus Candida is the mutation or/and overexpression of different genes (Whaley et al., 2017).

#### 2.1.1 Overexpression of the *ERG11* gene

Studies have shown that the overexpression of the *ERG11* gene is closely related to the resistance of *C. tropicalis* to azole drugs. By analyzing 507 clinical isolates, Fan et al. (2019) found that the expression level of *ERG11* in azole-resistant strains was significantly higher than that in sensitive strains, suggesting that the overexpression of this gene is directly involved in the resistance mechanism. Paul et al. (2022) further discovered that in drug-resistant strains without gene mutations, *ERG11* and its regulatory network (such as *CDR1*/*CDR3* efflux pump genes) showed systematic high expression, and the expression intensity was significantly higher than that of strains carrying mutations and sensitive strains. This overexpression phenomenon will lead to the production of the target enzyme (lanosterol 14α-demethylase) encoded by *ERG11* exceeding the inhibitory threshold of azole drugs, preventing the drugs from completely blocking the ergosterol synthesis pathway and ultimately forming a drug-resistant phenotype. The above two studies jointly confirm that the abnormal transcriptional regulation of *ERG11* is an important molecular mechanism for the development of azole resistance in *C. tropicalis*.

In addition, the research findings of Rojas indicate that for *C. tropicalis* strains exposed to fluconazole, regardless of their sensitivity characteristics, the expression levels of the *ERG11* gene are relatively high, followed by the *ERG3* and *MDR1* genes. In contrast, the relative expression level of the *CDR1* gene in *C. tropicalis* strains exposed to fluconazole is lower than that in strains not exposed to fluconazole (Rojas et al., 2023). These results may imply that drug exposure stimulation may lead to biological changes in *C. tropicalis* strains at the gene expression level, and this change has a significantly stronger impact on genes encoding target enzymes of the drug (such as the ERG family) than on genes encoding transporters related to drug efflux (such as the CDR family).

It is worth noting that the fluconazole resistance mediated by the overexpression of the *ERG11* gene in *C. tropicalis* may be related to the regulation of the zinc family transcription factor Upc2p. The analysis of 319 clinical strains by Wang D. et al. (2021) showed that the expression levels of *ERG11* and *UPC2* in the fluconazole-resistant group were significantly higher than those in the sensitive group, and there was a significant positive correlation between their expressions, suggesting that *UPC2* may positively regulate the transcription of *ERG11*. Paul et al. (2020) further verified this mechanism through *in vitro* induction experiments: when sensitive strains were passaged in a medium containing fluconazole, the expression level of *UPC2* increased by 12 times, accompanied by enhanced drug resistance. However, after withdrawing the drug, the expression of *UPC2* dropped back to a level close to the basal level, and the drug resistance decreased synchronously. These dynamic changes indicate that, as a core regulatory factor, *UPC2* drives the excessive synthesis of the target enzyme by upregulating the expression of *ERG11*, so that fluconazole cannot completely inhibit the enzyme activity, ultimately leading to the failure of drug target inhibition and the formation of a drug-resistant phenotype. The two studies jointly revealed the key role of the *UPC2-ERG11* regulatory axis in the acquired azole resistance of *C. tropicalis*.

In addition, Wan et al. (2025) developed a multi-SNP detection panel based on MALDI-TOF MS technology, which can identify drug-resistant phenotypes and provide support for the epidemiological study of drug resistance. The detection of 109 clinical isolates found that the high-frequency mutation sites were concentrated in G751A and A866T of the *UPC2* gene, A491T of the *TAC1* gene, and A395T and C461T of the *ERG11* gene. Among them, the mutations of G751A and A866T in *UPC2*, and A395T and C461T in *ERG11* significantly co-occurred. Further analysis showed that the mutations at the *ERG11*-395 and *ERG11*-461 sites were only present in azole-resistant strains, while the mutations at the *UPC2*-751, *UPC2*-866, and *TAC1*-491 sites were found in both drug-resistant and non-drug-resistant strains. Therefore, the co-occurring mutations of G751A/A866T in the *UPC2* gene may provide a genetic basis for the environmental adaptive evolution of *C. tropicalis*. In the future, attention should be paid to the co-evolution mechanism of the *UPC2*-*ERG11*-*TAC1* gene cluster to address the potential transmission risks of multidrug-resistant strains.

#### 2.1.2 Mutations of the *ERG11* gene

Mutations in the *ERG11* gene cause structural changes in the target enzyme of azole drugs (lanosterol 14α – demethylase), preventing azole drugs from binding to it and thus leading to drug resistance. The study by Siqueira et al. (2025) showed that the Y132F and Y257N mutations in *ERG11* are important mechanisms conferring resistance to azole drugs in *C. tropicalis*. In addition, after Jiang et al. (2024) processed the milk samples from 37 clinical cases of dairy cows with mastitis, an examination of bacterial virulence and pathogenicity in the PHI database revealed the presence of chemotherapeutic mutations at *CYP51*/*ERG11*. These mutations may further lead to the development of drug resistance.

##### 2.1.2.1 *ERG11* mutations and drug-resistant phenotypes

Some studies have analyzed the *ERG11* gene and found that the *ERG11* mutation A395T/W occurred in 10.7% (54/507) of the isolates, and all the *C. tropicalis* isolates with these mutations showed resistance to fluconazole (Fan et al., 2019). In addition, this study also reported for the first time that the V125A, Y257H, and G464S substitutions in *C. tropicalis* also conferred a fluconazole-resistant phenotype. The study by Ngo et al. (2023) showed that the missense mutations Y132F and S154F in the *ERG11* protein were associated with the resistance of *C. tropicalis* to fluconazole (accounting for 67.7%). In the study by Hu et al. (2023) on the main mechanisms of resistance to azole drugs in environmental and human commensal isolates of *C. tropicalis*, genomic analysis also indicated that the Y132F and S154F mutations in *ERG11* could confer the observed resistant phenotypes to these strains. It is worth noting that strains carrying both *ERG11* mutations and copy number variations have a high minimum inhibitory concentration (MIC≥16 μg/mL). In addition, phylogenetic analysis of 239 *C. tropicalis* strains identified 14 clades. Notably, all the isolates in the tenth clade were resistant to both fluconazole and voriconazole. Subsequently, through multilocus sequence typing (MLST) analysis, 239 *C. tropicalis* isolates from China could be divided into 132 diploid sequence types (DSTs), and the most common types were DST225, DST522, DST506, and DST346; among them, DST225 belonged to the tenth clade. The results of this study indicate that isolates carrying both *ERG11* hotspot mutations and genomic amplifications will show cross-resistance to fluconazole and voriconazole.

##### 2.1.2.2 The stable inheritance of drug-resistant clades

Tseng et al. (2024) analyzed the genetic correlations of *C. tropicalis* isolates and demonstrated that approximately 69% of the fluconazole dose-dependent sensitive isolates belonged to clade 4, among which 88.9% (8/9) belonged to DST225. Meanwhile, 9 out of 11 fluconazole-resistant strains (81.8%) also belonged to clade 4. The studies by Hu and Tseng successively mentioned the azole-resistant clades, indicating that the drug-resistant strains of *C. tropicalis* can stably inherit and express drug-resistance-related genes. Additionally, although the serial numbers of the drug-resistant clades are different, both clades contain the most common diploid sequence type DST225. From this, it can be inferred that these two so-called drug-resistant clades are essentially the same drug-resistant clade, and the two studies can further confirm each other. Moreover, Fan et al.’s (2024) research showed that *C. tropicalis* MLST clade 4 has become the dominant population resistant to azoles in the Asia-Pacific region. The typical characteristic of its subclade, the AZR resistance cluster, is the combination of tandem repeat amplification of the *ERG11* gene and the key A395T mutation. In addition, this special mechanism of copy number variation, together with the upregulation of the *ERG11* gene expression level, significantly increases the minimum inhibitory concentration (MIC) value of fluconazole. Meanwhile, the enrichment of Ty3/gypsy-like retrotransposons in the AZR resistance cluster enhances the genomic adaptability of the strain to environmental stresses, and the rapid spread of the AZR resistance cluster in China over the past few decades is the main reason for the sharp increase in the azole resistance rate (Fan et al., 2024).

In conclusion, the drug resistance of *C. tropicalis* is jointly driven by *ERG11* gene mutations, copy number variations, and the enrichment of transcription factors. Combined with the stable inheritance and adaptive evolution of drug-resistant clades, it has formed a multi-drug resistance mechanism and a transmission advantage against azole drugs.

##### 2.1.2.3 The mistranslation mutation of the *ERG11* gene

In addition, some studies have discovered and verified that three transformants with missense mutations in the *ERG11* gene, T769C, G1390A, and T374C, can lead to an increase in the minimum inhibitory concentration (MIC) of azole drugs by at least threefold (Fan et al., 2019). The conclusion of this study indicates that the missense mutation of *ERG11* is the main mechanism leading to azole resistance in *C. tropicalis* isolates. In contrast to the above, Khalifa et al. (2022) studied the azole resistance mechanisms and genotyping of 80 *C. tropicalis* strains (from clinical patients, animals, and the environment respectively). They observed that 83 nucleotide mutations were identified in the *ERG11* gene, most of which were synonymous mutations, and only 15 were nonsynonymous mutations leading to amino acid substitutions. Further comparison of the 15 missense mutations of *ERG11* showed that there were no missense mutations that led to drug resistance. However, the expression levels of the *TAC1*, *UPC2*, and *HMG* genes in the azole-resistant group of strains were significantly higher than those in the azole-sensitive group. The reason for this completely opposite result may be the complex diversity of organisms caused by different ecological niches. Based on phylogenetic analysis, this study also confirmed the cross-border and cross-transmission of *C. tropicalis* between humans and animals. According to the situation that the missense mutation of *ERG11* is not related to azole resistance, Paul et al. (2020) also indicated that no nonsynonymous mutations of *ERG11* were identified after inducing fluconazole resistance *in vitro*.

### 2.2 Enhancement of drug efflux

The efflux pumps related to drug resistance on the cell membrane of *C. tropicalis* include the ABC – series transporters encoded by the Candida drug – resistance *CDR1* gene and the major facilitator superfamily (MFS) encoded by the *MDR1* gene. The former obtains energy through ATP hydrolysis to actively excrete drugs across the cell membrane, while the latter does not require energy consumption.

Pandey et al. (2020) confirmed through quantitative PCR and sequence analysis that the genes of efflux pump transporters *CDR1*, *MDR1*, and *ERG11* were overexpressed in fluconazole – resistant strains of *C. tropicalis*. Moreover, the resistant strains carried specific mutations in *ERG11* (e.g., G1390A conferred the G464S amino – acid substitution, and A395T/C461T conferred the Y132F/S154F amino – acid substitutions respectively). This indicates that the overexpression of efflux pump transporter genes and *ERG11* mutations jointly mediate the development of fluconazole resistance in *C. tropicalis*. Khalifa et al. (2022) further found that the expression of *CDR2* and *CDR3* efflux pump genes and the transcription factors *TAC1* and *UPC2* was significantly up-regulated in drug-resistant strains. In conclusion, the resistance of *C. tropicalis* to azole drugs is jointly driven by *ERG11* gene mutations (e.g., Y132F, S154F), overexpression of efflux pump transporters (*CDR1*/*2*/*3*, *MDR1*), and up – regulation of transcription factors (*TAC1*, *UPC2*), forming a multi – drug resistance mechanism.

### 2.3 Alterations in the activity of the mitochondrial respiratory enzyme chain

The product encoded by the *CYTB* gene is cytochrome B in the mitochondrial respiratory chain. Fan et al. (2019) found that the expression level of the *CYTB* gene in the drug-resistant group of *C. tropicalis* was lower than that in the azole-sensitive group. According to the new research findings of Liu et al. (2024), the quantitative real-time polymerase chain reaction (QRT-PCR) method was used to determine the changes in the gene expression of mitochondrial respiratory chain enzymes, drug efflux pumps, and drug target enzymes in *C. glabrata* and *C. tropicalis* after treatment with butylphthalide (NBP, chemically named 3-n-butyl-1(3H)-isobenzofuranone, which is recorded to treat infections caused by microorganisms). The results showed that for *C. tropicalis*, after treatment with NBP, there were statistically significant differences in the gene expression of mitochondrial respiratory chain enzymes in azole-resistant *C. tropicalis* (Ct20) compared with the control group. Specifically, the gene expressions of mitochondrial respiratory chain enzymes *COX3* and *CYTB* decreased to 8.73 and 12.59% of the original levels, respectively. In contrast, the gene expressions of *COX1* and *COX2* increased to 2.25 times and 5.08 times of the initial values, respectively. Since mitochondrial respiratory chain enzymes are composed of mitochondrial complexes I–IV, they play different roles in the production of reactive oxygen species (ROS), and *COX1*, *COX2*, and *COX3* are the constituent subunits of mitochondrial respiratory chain enzyme complex IV. When specific subunits are overexpressed, the normal assembly of the complex will be affected, and the activity of the enzyme will also be affected (García-Villegas et al., 2017). Therefore, NBP exerts an antifungal effect on drug-resistant *C. tropicalis* by altering the activity of the mitochondrial respiratory enzyme chain.

In conclusion, since the study by Fan et al. (2019) indicates that the expression level of the *CYTB* gene in the drug-resistant group of *C. tropicalis* is lower than that in the azole-sensitive group, while the research results of Liu et al. (2024) show that NBP inhibits fungi by reducing the expression of genes such as *CYTB* in drug-resistant strains. There are discrepancies between the above two studies regarding the relationship between the expression of *CYTB* and the drug resistance of *C. tropicalis*. Therefore, the correlation between the alteration of the activity of the mitochondrial respiratory enzyme chain and the azole resistance of *C. tropicalis* still requires further research for verification. The following are the main ways in which *C. tropicalis* from different sources develops resistance to azole drugs (Table 1).

**Table 1:** Main ways of developing resistance to azole drugs.

Source of strains	Antifungal drugs	Genetic basis of drug resistance	Functional basis of resistance	References
Hospital patients	Azoles	Missense mutations in *ERG11*/overexpression of *ERG11;* downregulation of *CYTB.*	The affinity of lanosterol 14-*α*-demethylase for drugs is decreased/limited.	Fan et al. (2019)
National Centre for Pathogen Culture Collection, Chandigarh, India	Azoles	Nonsynonymous mutations in the *ERG11* gene.	The binding affinity of lanosterol 14-*α*-demethylase for drugs is decreased.	Paul et al. (2022)
Hospital patients	Azoles	Overexpression of *ERG11* and *UPC2*.	The concentration of lanosterol 14-α-demethylase increases.	Wang D. et al. (2021) and Gao S. et al. (2024)
National Pathogen Culture Collection Centre in Chandigarh, India	Azoles	Overexpression of *UPC2* (upregulating drug-resistant genes through the regulation of transcription factors).	The concentration of lanosterol 14-α-demethylase increases.	Paul et al. (2020)
Cow	Azoles	Mutations in *CYP51/ERG11* (There are nonsynonymous mutations in *CDR1*, *CDR2*, *CDR3*, *CDR4* and the ABC protein family).	Changes in drug transporters.	Jiang et al. (2024)
Environment, Clinic	Azoles	Mutations of Y132F and S154F in *ERG11*, and genomic amplification.	The binding affinity of lanosterol 14-α-demethylase for drugs is decreased.	Hu et al. (2023)
Orchard (papaya, apple, grape)	Azoles	Tandem gene duplication of the mutated *ERG11*.	The affinity of lanosterol 14-α-demethylase for drugs is decreased.	Tseng et al. (2024)
Patients, animals, environment	Azoles	Overexpression of *TAC1*, *UPC2* and *HMG* genes; Overexpression of *CDR2*, *CDR3* and *TAC1*.	Upregulation of drug transporters.	Khalifa et al. (2022)
Collection by the health care center	Azoles	Overexpression of *CDR1* and *MDR1*; Mutation of *ERG11*.	Upregulation of drug transporters; Decreased affinity of lanosterol 14-α-demethylase for drugs.	Pandey et al. (2020)

### 2.4 Resistance of biofilms

The matrix of mature biofilms contains various macromolecules, including proteins (55%), carbohydrates (25%), lipids (15%), and extracellular DNA (5%). These substances are one of the main factors contributing to the resistance and tolerance to antifungal drugs (Kaur and Nobile, 2023). In addition, the biofilm matrix not only supports the overall structure of the biofilm but also acts as a physical barrier to drug penetration. Some studies have shown (Marzucco et al., 2024) that among all Candida strains, compared with the planktonic form, the minimum inhibitory concentration (MIC) of the strains that form biofilms increases. Specifically, it is 86.8% (33/38) in *C. albicans*, 73% (19/26) in *C. parapsilosis*, 81.8% (9/11) in *C. glabrata*, and 87.5% (7/8) in *C. tropicalis*. This study further analyzed the statistical data and observed that especially in blood culture samples, the resistance of *C. tropicalis* (100%, belonging to the category of moderate biofilm-forming ability (MBF)) and *C. albicans* (75%, belonging to the categories of high biofilm-forming ability (HBF) and MBF) to fluconazole increased significantly, while the resistance of strains with low biofilm-forming ability (LBF) (*C. parapsilosis* and *C. glabrata*) increased only slightly (<50%). This indicates that Candida strains with stronger biofilm-forming ability have increased resistance to fluconazole. In addition, compared with planktonic cells in *C. tropicalis*, these biofilm cells show obvious differences in gene transcription, growth rate, and response to antifungal drugs. It is worth noting that the formation of biofilms makes fungal cells resistant to a variety of antifungal drugs (Dos Santos and Ishida, 2023; Malinovská et al., 2023).

In addition, the importance of matrix glucans and mannans in promoting the drug tolerance of biofilms appears to be a conserved mechanism, which is known to exist in the biofilms formed by various Candida species, such as *C. glabrata*, *C. parapsilosis*, *C. tropicalis*, and *Candida auris* (Dominguez et al., 2018; Dominguez et al., 2019). It is worth noting that Candida biofilms in the host environment are usually composed of multiple microorganisms and will form complex biofilms with many different species, which will ultimately change the composition of the matrix. For example, the amount of biofilm formed when *C. tropicalis* is cultured in a pre-formed *Staphylococcus epidermidis* biofilm (SE > CT group) is greater than that of single-microorganism (SE or CT) or mixed-microorganism (SE + CT) biofilms. At the same time, at 20 and 24 h of cultivation, the expression levels of icaB and icaC in the biofilm of the SE > CT group are higher than those of other groups, indicating an enhancement of matrix polymerization and transport, respectively (Phuengmaung et al., 2021). This may further change the drug isolation ability of these complex biofilms. For instance, in the study by Vila et al. (2021), *C. albicans* induced the activation of the biofilm formation network of *Staphylococcus aureus* by downregulating the autolysis inhibitory factor lrg operon, upregulating the ica operon, and increasing the production of polysaccharide intercellular adhesin (PIA), which, respectively, indicate an increase in the production of extracellular DNA (eDNA) and the extracellular polysaccharide matrix. This study further confirms that the increased production of extracellular DNA (eDNA) mediated by *C. albicans* in the mixed biofilm can play a role in the vancomycin tolerance of *S. aureus*. In the future, attention should be focused on whether there are different effects on the sensitivity of *C. tropicalis* to antifungal drugs when it forms complex biofilms with other microorganisms.

Recent studies have shown that Candida species release extracellular vesicles during the process of biofilm formation. These vesicles transport components of the extracellular matrix, including glucans and mannans, throughout the biofilm structure (Zarnowski et al., 2022). Interestingly, research has demonstrated that the proteins carried by individual vesicles can play a role in drug sequestration through the glucan-mannan complex of the extracellular matrix. The study by Zarnowski et al. (2022) identified a set of 36 proteins that are present in five species (*C. albicans*, *C. tropicalis*, *C. parapsilosis*, *C. glabrata*, and *C. auris*). Several of these proteins in this set have been studied in *C. albicans* and have been shown to have certain functions in biofilm development, suggesting that vesicle transport may be involved in their activities. Recent studies have also shown that other proteins in this set also have crucial functions in the biofilms of *C. albicans*, including biofilm dispersion and drug resistance. These proteins include Sun41, Cht3, and Tos1, etc. (Zarnowski et al., 2021). For example, compared with the complementary strains, the Tos1*Δ*/Δ strain (the Tos1-deficient strain is used to evaluate the sensitivity of biofilms to fluconazole) shows higher sensitivity to antifungal drugs in *C. albicans*, *C. tropicalis*, *C. parapsilosis*, *C. glabrata*, and *C. auris* (Zarnowski et al., 2022; Zarnowski et al., 2021). In addition, some studies have claimed that extracellular vesicles may be related to the resistance of *C. tropicalis* biofilms to fluconazole and caspofungin (Kulig et al., 2022). These findings indicate that extracellular vesicles play an important role in promoting the drug resistance of Candida biofilms and may become a new target for future antifungal drug therapies.

In addition, Jeyarajan’s study reported for the first time that the epinecidin-1 variants modified by0 lysine substitution have anti-Candida and anti-biofilm effects on multidrug-resistant clinical isolates. Compared with the wild-type epinecidin-1, the activities of these variants have increased by 2 to 8 times, and they have shown significant efficacy in disrupting the integrity of the fungal cell membrane and inhibiting biofilm formation (Jeyarajan et al., 2024). This study has confirmed that such polypeptides have potential value for application as anti-biofilm agents on the surface of medical implants.

## 3 Resistance mechanisms to echinocandins

With the development of fungal resistance to azoles, echinocandins have become widely used drugs in clinical practice. Echinocandins (such as anidulafungin, micafungin, and caspofungin, etc.) act on the biosynthesis of (1,3)-*β*-d-glucan synthase encoded by the *FKS1* and *FKS2* genes, thus preventing the correct synthesis of glucan and leading to the loss of cell wall integrity. As a result, echinocandins have a fungicidal effect on most Candida species. Although resistance of *C. tropicalis* to echinocandins is not common, there have been successive reports on the development of echinocandin resistance in *C. tropicalis* (Favarello et al., 2021; Boattini et al., 2023; Baniodeh et al., 2024). In addition, some studies have observed that, compared with the period before the COVID-19 pandemic, the MIC50 and MIC90 values of *C. tropicalis* to azoles (fluconazole, itraconazole, and posaconazole) and echinocandins (anidulafungin) have increased to varying degrees during the COVID-19 outbreak (Szekely et al., 2023).

The decrease in echinocandin resistance or the decline in susceptibility is mainly due to mutations in the highly conserved regions of the *FKS* genes, and the level of resistance depends on the hotspot mutations and expression levels of these genes. Sfeir et al. (2020) performed DNA sequencing of two hotspot (HS) regions of the drug target gene *FKS1* in three clinical isolates of *C. tropicalis* (BL37986, BL38734, and isolate 2). These two hotspot regions are known to confer echinocandin resistance. Sequence analysis showed that in the BL38734 strain, a heterozygous T-C mutation occurred at position 654 of HS1, resulting in the substitution of serine with proline, and the same mutation was also found in isolate 2. Four years later, the study by Yang et al. (2024) once again confirmed that the resistance of *C. tropicalis* strains to echinocandins is associated with the S654P variation in the hotspot region of the *FKS1* gene. It is worth noting that in one isolate of *C. tropicalis*, in addition to the S654P variation in the *FKS1* gene, there was also an R1220T variation. And a case with the S654P variation plus the G324R variation in the hotspot region of the *FKS1* gene was also found. These data indicate that when strains are exposed to antifungal drugs, it will prompt them to continuously update their escape mechanisms from antifungal drugs, which in turn leads to a general increase in the resistance of strains to antifungal drugs. Lim et al. (2020) evaluated the resistance of Candida isolates to azoles and echinocandins through the new Vitek 2 AST-YS08 (YS08) and Sensititre Yeast One (SYO) systems. Among them, 24 were *C. tropicalis* (10 *ERG11* and 1 *FKS* mutants). According to the clinical case reported by Xiao et al. (2018), an echinocandin-resistant *C. tropicalis* isolate was isolated and cultured from the pleural drainage fluid of a 60-year-old female patient with severe coronary artery disease and pulmonary infection. Further research showed that a mutation leading to the amino acid substitution S80P was found in the HS1 region of the *FKS1* gene of the *C. tropicalis* strain 13TJ350.

By analyzing the above studies, we can observe that echinocandin-resistant *C. tropicalis* has a common characteristic: there are abnormalities in the *FKS* gene. In addition, according to the studies by Sfeir et al., Yang et al., and Xiao et al., the resistance of *C. tropicalis* to echinocandins may be achieved due to the amino acid substitution in the HS1 region of the *FKS1* gene. This will lead to the failure of the target that echinocandins can act on, preventing it from stopping the correct synthesis of glucan and thus failing to exert its antibacterial effect.

In addition, in a recent study, researchers isolated *Wickerhamiella tropicalis* from the blood sample of a 6-year-old girl with a history of B-cell precursor lymphoblastic leukemia in Japan in 2022. Although through routine microbiological examination, this strain was morphologically identified as a species of the genus Candida, it was subsequently determined to be *W. tropicalis* by sequencing the internal transcribed spacer (ITS) region of ribosomal DNA (rDNA) (Takei et al., 2024). Interestingly, this isolate had amino acid substitutions in the *ERG11* and *FKS1* genes, which are related to azole and echinocandin resistance, respectively. Such substitutions made this strain show moderate resistance to fluconazole and micafungin. This further indicates that amino acid substitutions in the *FKS* gene can lead to the resistance of the genus Candida to echinocandins.

## 4 The resistance mechanism to polyene drugs

Classical polyene antifungal drugs, such as amphotericin B (AMB), are characterized by a broad antibacterial spectrum and high antibacterial activity. They are mainly used to treat severe systemic fungal infections. However, there are occasional reports of resistance of *C. tropicalis* to AMB (Forastiero et al., 2013; Pasrija et al., 2024; Biswas et al., 2023). AMB interacts with ergosterol in the fungal plasma membrane. It achieves the antibacterial effect by forming pores to trigger cellular ion leakage or extracting ergosterol from the plasma membrane, which leads to cell death. The resistance to AMB is mainly caused by changes in the content or structure of ergosterol.

### 4.1 Ergosterol deficiency

Eddouzi et al. (2013) conducted a study and found that the isolated strain of *C. tropicalis* (JEY162) exhibited cross-resistance to fluconazole (FLC), voriconazole, and amphotericin B. By analyzing sterols using gas chromatography (GC)-mass spectrometry (MS), it was discovered that ergosterol was absent in JEY162, while 14α-methyl non-sterols accumulated. This revealed that the functions of the key proteins ERG11 and ERG3 in ergosterol biosynthesis were disrupted. However, these two alleles were confirmed to be non-functional, which is consistent with previous research conclusions that the *ERG11* mutant must coexist with other *ERG3* mutations for survival. In addition, some studies have also shown that multidrug-resistant *C. tropicalis* strains with double mutations of *ERG11* and *ERG3* are resistant to both amphotericin B and azole drugs (Sharma and Chakrabarti, 2023). However, by replacing the defective genes in JEY162 with the wild-type alleles of *CtERG3* and *CtERG11*, the same drug-resistant phenotype as that of JEY162 was generated, indicating that these mutations are involved in the development of drug resistance. Moreover, during the process of reconstructing drug resistance, a strain carrying only the defective *CtERG11* allele was obtained. Its main sterol was the toxic metabolite 14α-methyl ergosta-8,24(28)-diene-3α,6β-diol, which suggests that *ERG3* is still functioning and challenges the view that the *ERG11* mutation must be accompanied by a compensatory mutation for survival (Eddouzi et al., 2013). In conclusion, the analysis of the above results confirms that the activity defects of sterol 14α-demethylase and sterol Δ5,6-desaturase in clinical *C. tropicalis* lead to azole-polyene cross-resistance.

The study by Forastiero et al. (2013) sequenced the *ERG3* gene of drug-resistant strains, which showed that there were single missense mutations C773T and A334G in the *ERG3* sequences of ATCC 200956 and CL-6835 respectively, leading to amino acid substitutions of S258F and S113G. The sterol profiles of sensitive and drug-resistant strains were analyzed by gas chromatography (GC)-mass spectrometry (MS). In contrast, ergosterol was not detected in any of the AMB-resistant strains. The results of the Rhodamine 6G (R6G) test showed that all strains captured R6G, and the amphotericin B-resistant strains exhibited a higher uptake or release ratio than the amphotericin B-sensitive strains at time zero. This phenomenon can be explained by the higher membrane permeability caused by changes in the composition of membrane sterols. In addition, protein sequencing and structural analysis showed that strain ATCC 200956 lacked 44 amino acids in ERG11p, which corresponded to the excision of the “I” helix in the azole target. This deletion may render the protein non-functional, which can explain the deficiency of ergosterol and the accumulation of 14α-methylated sterols, thus accounting for the resistance to amphotericin B.

Huang (2014) induced drug-resistant strains *in vitro* by using the method of increasing concentrations of fluconazole (FCZ) and AMB, and detected the expression levels of drug-resistant genes in wild strains and drug-resistant strains by real-time fluorescence quantitative PCR. It was found that the overexpression of genes such as *CYP51A*, *CYP51B*, *CDR1*, *CDR2*, *MDR1*, *MDR2*, *Yap1*, and *Sho1* was related to the resistance of *C. parapsilosis* and *C. tropicalis* to AMB. The overexpression of genes such as *CYP51A*, *MDR1*, *CDR1*, *CDR2*, *Yap1*, and *Shol* was related to the resistance of *C. parapsilosis* and *C. tropicalis* to FCZ. This reveals that after long-term exposure of *C. parapsilosis* and *C. tropicalis* to FCZ and AMB, it can induce the overexpression of genes of key enzymes in the ergosterol synthesis process, efflux pump genes, and signal transduction-related genes, thus leading to the development of drug resistance.

### 4.2 Changes in the biofilm

The formation of biofilm is an important virulence factor of Candida species, and some studies have shown that biofilm is related to the drug resistance of Candida. According to the report by Perlin et al. (2017), biofilms have genetic resistance to amphotericin B and fluconazole both clinically and in vitro. This genetic resistance of biofilms provides a shelter for microorganisms and the opportunity to withstand high concentrations of antifungal agents. The study by Fernandes et al. (2015) showed that AMB could not completely prevent the formation of biofilms, nor could it eradicate the pre-formed biofilms of *C. tropicalis*. The obvious increase in the content of proteins and carbohydrates in the biofilm matrix treated with AMB seems to be the main reason for the enhanced drug resistance of *C. tropicalis* biofilms. As Meng et al. (2017) found in their study, it was confirmed that the biofilm matrix of *C. tropicalis* is rich in hexosamine, making it difficult for antifungal drugs to penetrate. Therefore, the biofilm of *C. tropicalis* develops resistance to amphotericin B by synthesizing a large amount of matrix rich in hexosamine.

### 4.3 Changes in the components of the cell wall

Mesa-Arango et al. (2016) described that amphotericin B-resistant strains showed basal activation levels of *Mkc1*, *Hog1*, and *Cek1 MAPKs*, while sensitive strains did not, which may be related to the changes in the cell wall components of the resistant strains. Through research, it was found that the high β-1,3-glucan component in the AMB-resistant strains may inhibit the permeation of amphotericin B into the cells. β-1,3-glucan is synthesized by β-1,3-glucan synthase on the membrane. The decrease in the ergosterol component on the membrane of the resistant strains may lead to the alteration of the activity of β-1,3-glucan synthase, resulting in the production of high levels of β-1,3-glucan. However, currently, due to the limitations of molecular biology tools, it is impossible to determine whether this change is directly related to the resistance to amphotericin B.

## 5 The resistance mechanism to flucytosine

5-Fluorocytosine is a fluorinated pyrimidine analog with antibacterial activity. It enters the cell through the cytosine permease on the fungal cell membrane, is converted into 5-fluorouracil by cytosine deaminase, and then is converted into 5-fluorouridine monophosphate or 5-fluorodeoxyuridine monophosphate by UMP pyrophosphorylase, thereby inhibiting the synthesis of proteins and DNA (Campoy and Adrio, 2017). Although 5-fluorocytosine shows excellent antibacterial activity against most Candida species, in the study by Chen et al. (2011), 30 sensitive isolates could produce drug-resistant offspring after exposure to this drug. Additionally, Charlier et al. (2015) observed a high rate of acquired resistance to this drug during treatment with a single drug. Through the analysis of the above research results, the phenomenon that 5-fluorocytosine is prone to develop drug resistance may lead to the limitation of the use of this drug. Recently, some studies have reported that the isolated strains of *C. tropicalis* also show slight resistance to flucytosine (Keighley et al., 2024; Delma et al., 2024). In addition, a study from southern India showed that the resistance rate of *C. tropicalis* to flucytosine was 77.46% (*n* = 55) (Umamaheshwari and Sumana, 2023). There is no doubt that the reason for this phenomenon is the increased drug resistance caused by the frequent and unselective use of flucytosine.

Acquired resistance to flucytosine is due to enzymatic mutations leading to metabolic disorders. Specifically, the resistance to 5-fluorocytosine is related to the K177E mutation of the URA3 gene. The Ura3 enzyme is involved in the metabolic pathway of uracil monophosphate (UMP), and UMP is a substrate for thymidylate synthase and UMP kinase, both of which are involved in nucleic acid synthesis. The study by Desnos-Ollivier et al. (2008) showed that the 5-fluorocytosine-resistant strains of *C. tropicalis* had a deletion of A at position 106 in the ITS2 region, that is, a K177E missense mutation occurred in the *URA3* gene. Although some 5-fluorocytosine-sensitive strains also have this mutation, when additional genotypic markers are used, differences are found between 5-fluorocytosine-sensitive strains and 5-fluorocytosine-resistant strains. In addition, Chen et al. (2011) observed changes in the amino acid sequence of the *URA3* gene in drug-resistant *C. tropicalis* strains. In this study, 30 sensitive strains could produce drug-resistant offspring after drug exposure. It is worth noting that at position 145 of the *FCY2* gene (encoding purine-cytosine permease) in 22 clinical isolates, there was a G/T heterozygous state, while in the offspring strains recovered from the inhibition ring, this position changed to a T/T homozygous type. This mutation results in the generation of null alleles in both gene copies, and only truncated proteins can be expressed, thus leading to resistance to 5FC.

So far, only four classes of antifungal drugs can be used to treat systemic fungal infections: azoles and polyenes, which act on the fungal membrane level; echinocandins, which act on the fungal cell wall; and flucytosine, which interacts with nucleic acid synthesis. However, they have all shown varying degrees of drug resistance. Therefore, it is urgent to find alternatives that can address the drug resistance of *C. tropicalis*.

## 6 Solutions strategies

The study indicates that the sharp increase of drug-resistant *C. tropicalis* in the environment is closely related to the use of azole drugs. The extensive use of azole fungicides in the agricultural field is one of the reasons for the growth of drug-resistant bacteria. The fluconazole-resistant *C. tropicalis* strains isolated from orchards in Taiwan are genetically similar to the strains causing human infections, suggesting that the increase of drug-resistant *C. tropicalis* in the environment may be related to the frequent use of drugs in clinical settings and the massive application of azole fungicides in agriculture (Khalifa et al., 2022). In addition, Chen et al. (2023) have confirmed through research that fruits can serve as carriers of azole-resistant *C. tropicalis*. It is worth noting that besides being found in patients, in this study, the researchers also identified azole-resistant *C. tropicalis* with the dominant genotype of clade 4 from fruits. However, it remains to be studied whether the original azole-resistant *C. tropicalis* of clade 4 originated from patients taking azole drugs in clinical settings, from the environment where agricultural fungicides are used, or from both. According to the concept of “one health” (Castelo-Branco et al., 2022), the antifungal management work in both human hospitals and veterinary hospitals can help reduce the selection of drug-resistant bacteria. However, if the agricultural field does not take corresponding measures to reduce or stop the use of fungicides of the same type as those used in medicine, then susceptible patients will still continue to be infected by highly drug-resistant bacteria, and the treatment options will be extremely limited.

Based on this, in addition to more reasonable control of the dosage and types of drugs used in clinical settings, we should also invite relevant agricultural departments to formulate practical policies to reduce or stop the use of fungicides used in the medical field. At the same time, efforts should be made to increase the research and development of new antifungal drugs. The following reviews the studies on the extracts of some natural spice plants and chemical substances against drug-resistant *C. tropicalis*, and also summarizes a potentially neglected antifungal signaling pathway with great potential.

### 6.1 Research on the extracts of natural spice plants against drug-resistant *Candida tropicalis*

Taveira et al. (2016) found in their research that the thionin-like peptide (CaThi) purified from the fruits of *Capsicum annuum* has strong bactericidal activity against six pathogenic Candida species, including *C. tropicalis*. It exerts its effect by permeating the cell membrane and inducing an oxidative stress response, and there are nuclear targets within the cells of *C. tropicalis*. Observations under an optical microscope showed that the combined use of CaThi and fluconazole (FLC) can significantly change the morphology of yeast cells, and it is effective against all tested Candida species. The combined treatment of the two is expected to improve the therapeutic effect on drug-resistant strains of Candida species.

Thionin-like peptides, with their broad-spectrum antibacterial activity, multi-target action mechanism, and the advantage of being of natural origin, have become a highly promising raw material for lead compounds against drug-resistant microorganisms (Afroz et al., 2020). However, as small molecular peptides (approximately 5 kDa), thionin-like peptides face the problems of poor *in vivo* stability (easily degraded by proteases) and low bioavailability. In addition, the toxicity of thionin-like peptides is the main limitation for their clinical application. Therefore, for their clinical translation, it is necessary to break through the bottlenecks of toxicity and bioavailability, and achieve a “safety-efficacy” balance through structural optimization, innovation of delivery systems, and combination therapy strategies. In the short term, the treatment of local infections (such as topical preparations) is a more feasible application direction. In the long term, it is necessary to combine synthetic biology and precision medicine technologies to develop highly selective derivatives, making it possible to transform plant defense molecules into novel antibacterial agents in the fields of human health and agriculture.

Liu et al. (2024) showed in their research that the active monomer N-butylphthalide (NBP) isolated from celery seeds has antifungal activity against *C. glabrata* and *C. tropicalis*. Its mechanism of action is to affect the gene expression of mitochondrial respiratory chain enzymes, reverse the high expression of drug efflux pump genes CDR1 and CDR2 related to drug resistance, thereby enhancing the antibacterial activity of fluconazole against drug-resistant Candida species. It was further found that the targets of NBP are mitochondrial respiratory chain enzyme complexes III (*CYTB*) and IV (*COX1*, *COX2*, *COX3*), providing new potential targets for the development of antifungal drugs.

The NBP has antifungal activity against both drug-resistant and sensitive Candida species. However, due to its relatively high minimum inhibitory concentration (MIC) value, its *in vivo* application may be limited. Therefore, in clinical practice, its potential benefits against invasive Candida infections may be limited. However, as a natural plant extract, NBP may have potential development value as an *in vitro* therapeutic drug. As a drug that has been used for a long time in clinical practice, NBP has few adverse reactions and good safety. In addition, it also exhibits certain anti-inflammatory and immunomodulatory effects, which may be achieved by reducing oxidative stress and regulating the nuclear factor kappa-light-chain-enhancer of activated B cells (NF-κB) pathway (Wang B. N. et al., 2021; Li et al., 2020).

In their research, Pasrija et al. (2024) used commercially available essential oil of Zanthoxylum planispinum and determined the antibacterial situation through the minimum inhibitory concentration (MIC) and agar diffusion method. The results showed that it exhibited significant antifungal activity against various Candida species. Further research revealed that the antifungal activity of the prickly ash oil was fungicidal and involved reducing the ergosterol level in the cell membrane.

Currently, there is a lack of toxicity data for the essential oil of Zanthoxylum planispinum. It is necessary to conduct safety evaluations (such as in vitro and in vivo toxicity tests and the risk of long-term exposure) to determine the safe dosage range. Most of the components of the essential oil are lipophilic small molecules (such as terpenes, aldehydes, and ketones), and when taken orally, they may be affected by gastrointestinal metabolism and the first-pass effect, resulting in relatively low bioavailability. It is necessary to optimize the formulation (such as using nanocarriers and lipid delivery systems) to increase its concentration at the target site, and at the same time, conduct pharmacokinetic studies. If the above-mentioned studies achieve positive results, the essential oil of Zanthoxylum planispinum is expected to become a supplement to antifungal drugs (especially for superficial infections or combination therapy), but it still requires long-term systematic research support.

The research findings of Lemos et al. (2020) demonstrated for the first time that scopoletin, a coumarin isolated from *Mitracarpus frigidus* (*M. frigidus*), exhibits antifungal activity against the clinically relevant multidrug-resistant *C. tropicalis* ATCC®28,707 strain. Further investigation into its mechanism of action revealed that scopoletin inhibits the growth of microorganisms and induces cell death by interfering with the synthesis of fungal cells and disrupting the cell wall and plasma membrane, which provides preliminary insights into its bacteriostatic and bactericidal processes. Additionally, scopoletin affects the growth rate of pre-formed biofilm of *C. tropicalis* as well as the stages of its formation and proliferation. Therefore, the current data support the development of drugs based on plant-isolated scopoletin for the treatment of candidiasis caused by *C. tropicalis*.

Studies have shown that scopoletin has a low bioavailability, but it is rapidly absorbed and extensively metabolized, and it is non-toxic to most of the tested cell types (Gao X. Y. et al., 2024). Taking into account its advantages and limitations, scopoletin is a suitable lead compound for the development of novel derivatives with high efficiency and low toxicity. However, further research is still needed to explore its molecular mechanism and targets of action, verify its toxicity, and enhance its oral bioavailability.

### 6.2 Research on chemical substances against drug-resistant *Candida tropicalis*

Contreras-Martínez et al. (2023) conducted a comprehensive transcriptomic analysis of *C. tropicalis* exposed to isoxylopic alcohol (ISO). ISO is a monoterpene derived from Oxandra xylopioides. The researchers utilized transcriptomic techniques with the aim of revealing the complex transcriptional changes induced by ISO in *C. tropicalis*. Through differential gene expression analysis, it was shown that 186 genes responded to ISO, with 85% of them being upregulated. These upregulated genes are involved in key processes such as ergosterol synthesis, protein folding, and the DNA damage response, while 27 downregulated genes affect cytoplasmic translation, membrane proteins, and so on. This indicates that ISO can act on multiple important pathways within *C. tropicalis*, reflecting the complexity of the antifungal mechanism of ISO, and it is a potential candidate drug against drug-resistant *C. tropicalis*.

Although there is no clear data reported on the bioavailability of ISO. However, studies have confirmed that within the evaluated dose range, ISO did not exhibit cytotoxicity and could enhance the efficacy of classical antifungal drugs through a synergistic effect (Contreras-Martínez et al., 2024). This characteristic makes it a monoterpene compound with significant value for pharmacological development, providing a dual strategy for dealing with drug-resistant Candida infections – it can be used not only as a low-toxic candidate drug alone but also as a potentiator for existing therapies.

Lo et al. (2020) found that the treatment with chitosan combined with fluconazole exhibited excellent synergistic bactericidal effects against *C. albicans* and *C. tropicalis*. However, when chitosan was combined with amphotericin B or chitosan was combined with caspofungin for bactericidal purposes, there was no difference in the impact on antifungal activity. These findings provide strong evidence indicating that the combination of chitosan and fluconazole is a promising treatment approach for the two species of Candida and their drug-resistant strains. This also reminds us that when using drugs clinically, while paying attention to the synergistic effects of drugs, we should not overlook the influence brought about by the antagonistic effects.

Although chitosan has excellent biological properties such as biocompatibility, biodegradability, non-toxicity, relatively high bioavailability, and strong antibacterial properties, a great deal of research is still required to overcome the obstacles to clinical translation and transform chitosan into a novel antifungal agent.

Some studies have shown (da Silva et al., 2022) that two types of AuNPs (gold nanoparticles) affect the growth of biofilms through the accumulation of reactive oxygen species and reactive nitrogen intermediates, thereby exhibiting bactericidal activity and cellular stress. Compared with the planktonic form, biofilms are at least 100–1,000 times more resistant to the action of antibacterial drugs. The emergence of nanoparticles provides a new method for improving the safety and effectiveness of antibacterial treatment.

Gold nanoparticles can serve as an excellent delivery system to enhance the bioavailability of target drugs, which indicates that they have good bioavailability themselves (Kumar Seetharaman et al., 2023). However, some studies have shown that AuNPs may have toxic effects at the cellular, tissue, and organ levels (Niżnik et al., 2024). Considering both their advantages and limitations, further research is needed to explore their molecular mechanisms and targets of action, reduce their toxicity, and maintain the characteristic of good bioavailability. Only in this way can gold nanoparticles be transformed into highly effective, non-toxic antifungal drugs with high bioavailability.

### 6.3 Potential signaling pathways against drug resistance in *C. tropicalis*

In the face of the severe challenge of drug resistance of *C. tropicalis* in clinical practice, and because fungi are eukaryotes, there are limited targets for designing drugs with low toxicity and high efficiency. Moreover, the research and development of new antibacterial drugs require huge economic investment, a strong research team, and will take many years. Therefore, combination therapy has become one of the effective strategies to overcome fungal drug resistance nowadays. Some studies have shown that the combination of isavuconazole and amphotericin B can successfully treat disseminated *C. tropicalis* infections (Teng et al., 2024).

In recent years, the research on the calcium signaling pathway in fungal cells has gradually become an important direction for exploring the mechanism of fungal drug resistance. As a key protein in the calcium signaling pathway of *C. albicans*, calcineurin plays a core role in maintaining the balance of calcium ions in fungal cells. The activated calcineurin ensures the normal growth of fungal cells by finely regulating the concentration of calcium ions. Existing studies have confirmed that this protease is closely related to the virulence of fungi in the blood and their tolerance to drugs (Zhang et al., 2012). A large number of studies have shown that some non-antifungal drugs that can interfere with the functions of the components of the calcium signaling pathway in fungal cells, when used alone or in combination with fluconazole, can combat fungal drug resistance (Liu S. et al., 2015). This is undoubtedly an effective way to overcome fungal drug resistance and also provides new clues and ideas for the development of new drugs.

Taking the research of Liu S. Y. (2015) as an example, they deeply explored the effects and mechanisms of the combination of calcium channel blockers and fluconazole against drug-resistant *C. albicans*. The research results showed that the combination of fluconazole and calcium channel blockers had a strong synergistic effect on drug-resistant *C. albicans*. Specifically, calcium channel blockers such as amlodipine (AML) and nifedipine (NIF) at 8 μg/mL, and benidipine (BEN) and flunarizine (FNZ) at 16 μg/mL significantly reduced the minimum inhibitory concentration (MIC) of fluconazole against drug-resistant strains, greatly decreasing it from 512 μg/mL to 1 μg/mL or 2 μg/mL. Furthermore, taking AML, which has the best synergistic effect with fluconazole (FLC) among the calcium channel blockers, as the research object, fluorescence probes and flow cytometry technology were used to monitor the changes in the intracellular calcium ion concentration of drug-resistant *C. albicans* cells after treatment with AML alone or in combination with FLC. The results showed that only the combination of AML and FLC could significantly increase the intracellular calcium ion concentration. This phenomenon reveals that the synergistic mechanism of the combination of AML and FLC is most likely closely related to disrupting the intracellular calcium ion balance of fungal cells.

To deeply explore the mechanism of action, the research team further studied the effect of the combination of FLC and calcium channel blockers on the expression of calcium channel-related genes in drug-resistant *C. albicans* cells. Similarly, AML, which has the best synergistic effect with FLC, was selected as the test drug. The total RNA of drug-resistant *C. albicans* treated with the two drugs alone or in combination was extracted, and the expression levels of genes such as *CCH1*, *MID1*, *CNB1*, and *YVC1* in the calcium signaling pathway were accurately determined by ordinary PCR and real-time quantitative PCR. The results showed that only the combination group could cause a decrease in the expression levels of *CNA1*, *CNB1*, and *YVC1*, among which the decrease in the expression level of *CNA1* was the most significant. This decrease in expression may inhibit the regulation of the intracellular calcium ion balance in fungal cells by weakening the activity of calcineurin. At the same time, the calcium channel encoded on the vacuolar membrane that allows intracellular calcium ions to enter the vacuole is inhibited, blocking the vacuole’s uptake of the increased calcium ions. This leads to a continuous increase in the intracellular calcium ion concentration, a severe disruption of the calcium balance, and ultimately results in cell death.

In addition, the research by Li et al. (2021) revealed the possibility of the calcineurin signaling pathway as a potential drug target for treating *C. albicans* from multiple dimensions. It specifically involves proteins that regulate the intracellular Ca^2+^ concentration, proteins that sense changes in the intracellular Ca^2+^ concentration, downstream effectors of the calcineurin signaling pathway, and proteins that stabilize the calcineurin signaling pathway. Notably, calcineurin also plays a crucial role in *C. tropicalis*, being able to control its drug tolerance. In a mouse model of systemic infection, studies have confirmed that calcineurin can regulate the hyphal growth of *C. tropicalis* (in response to carbon source starvation), virulence, and drug tolerance to micafungin, and these functions are partially dependent on *Crz1* (Chen et al., 2014). This fully demonstrates that calcineurin has an indispensable position in the drug tolerance of Candida species, making it highly likely to become an important drug target for *C. tropicalis*.

Currently, some innovative drugs are still in the clinical trial stage, and their effectiveness and safety in the human body still need to be further verified. With the continuous in-depth research, it is expected that these drugs will be approved for clinical treatment in the future, which may potentially solve the problem of drug resistance in clinical practice. In addition, the research on the relationship between the calcineurin signaling pathway and *C. tropicalis* is relatively scarce. Given the crucial role of the calcineurin signaling pathway in the physiological processes of fungi, it is urgent to deeply explore its associated mechanism with *C. tropicalis*. By strengthening research in this area, it is hoped that key proteins or molecules in the calcineurin signaling pathway can be used as drug targets to develop innovative drugs against *C. tropicalis* infections, thereby providing more effective means for the clinical treatment of *C. tropicalis* infections.

## 7 Summary

The increasing drug resistance of *C. tropicalis* has made clinical treatment, especially the treatment of invasive infections, more difficult. The drug resistance to azole drugs is mainly related to the mutations of specific genes and the increase in the expression levels. In recent years, *C. tropicalis* strains resistant to echinocandins, polyenes, and 5-fluorocytosine have also been discovered, but the research on these resistance mechanisms is still insufficient. The drug resistance mechanism of *C. tropicalis* is relatively complex, and the interactions and influences among different resistance mechanisms still need further study. In this situation, exploring new treatment strategies, opening up new treatment pathways, and searching for new therapeutic targets are of great significance for overcoming drug resistance and improving the clinical cure rate.

Compared with previous reviews, this review not only covers the latest research achievements but also puts forward the hypothesis of “stable inheritance of drug-resistant lineages” for the first time. The drug resistance of *C. tropicalis* is jointly driven by the mutation of the *ERG11* gene, copy number variation, and the enrichment of transcription factors. Combined with the stable inheritance and adaptive evolution of drug-resistant lineages, a multiple drug resistance mechanism to azole drugs and a transmission advantage have been formed. Moreover, the sources of drug-resistant strains of *C. tropicalis* are further classified (whether the drug-resistant strains are from humans, the environment, or animals), and in combination with the concept of “one health,” all parties are called upon to abide by the principles of using fungicides (that is, reducing or stopping the use of fungicides of the same type as those in medicine). In addition, the research on some potential natural extracts and chemical substances against drug-resistant *C. tropicalis* is reviewed and critically commented on, aiming to provide ideas for solving the increasingly serious drug resistance problem of *C. tropicalis*. In addition, through a comprehensive analysis of a large number of literatures, this review comprehensively summarizes for the first time the structural and functional differences of calcineurin among different Candida species, and how these differences affect drug tolerance, providing an important theoretical basis for the development of specific treatment strategies for *C. tropicalis*. In addition, due to the numerous difficulties faced by new antifungal drugs from research and development to clinical application, and the increasing drug resistance of clinical antifungal drugs year by year, this study mentions that combination therapy has become one of the key strategies to overcome current fungal drug resistance.
